# Rescue radiofrequency ablation or percutaneous ethanol injection: a strategy for failed RALPPS stage-1 in patients with cirrhosis-related hepatocellular carcinoma

**DOI:** 10.1186/s12893-021-01241-z

**Published:** 2021-05-18

**Authors:** Qiang Wang, Shu Chen, Jun Yan, Torkel Brismar, Ernesto Sparrelid, Chengming Qu, Yujun Ji, Shihan Chen, Kuansheng Ma

**Affiliations:** 1 Division of Medical Imaging and Technology, Department of Clinical Science, Intervention and Technology(CLINTEC), Karolinska Institutet, Stockholm, Sweden; 2 Institute of Hepatobiliary Surgery, Southwest Hospital, Third Military Medical University (Army Medical University), No. 30 Gaotanyan Main Street, Shapingba District, Chongqing, 400038 China; 3 Division of Surgery, Department of Clinical Science, Intervention and Technology (CLINTEC), Karolinska Institutet, Karolinska University Hospital, Stockholm, Sweden

**Keywords:** Associating liver partition and portal vein ligation for staged hepatectomy (ALPPS), Radiofrequency ablation (RFA), Percutaneous ethanol injection (PEI), Hepatocellular carcinoma (HCC), Future liver remnant (FLR)

## Abstract

**Background:**

The future liver remnant (FLR) faces a risk of poor growth in patients with cirrhosis-related hepatocellular carcinoma (HCC) after stage-1 radiofrequency-assisted ALPPS (RALPPS). The present study presents a strategy to trigger further FLR growth using supplementary radiofrequency ablation (RFA) and percutaneous ethanol injection (PEI).

**Methods:**

At RALPPS stage-1 the portal vein branch was ligated, followed by intraoperative RFA creating a coagulated avascular area between the FLR and the deportalized lobes. During the interstage period, patients not achieving sufficient liver size (≥ 40%) within 2–3 weeks underwent additional percutaneous RFA/PEI of the deportalized lobes (rescue RFA/PEI) in an attempt to further stimulate FLR growth.

**Results:**

Seven patients underwent rescue RFA/PEI after RALPPS stage-1. In total five RFAs and eight PEIs were applied in these patients. The kinetic growth rate (KGR) was highest the first week after RALPPS stage-1 (10%, range − 1% to 15%), and then dropped to 1.5% (0–9%) in the second week (p < 0.05). With rescue RFA/PEI applied, KGR increased significantly to 4% (2–5%) compared with that before the rescue procedures (p < 0.05). Five patients proceeded to RALPPS stage-2. Two patients failed: In one patient the FLR remained at a constant level even after four rescue PEIs. The other patient developed metastasis. Except one patient died after RALPPS stage-2, no severe complications (Clavien-Dindo ≥ IIIb) occurred among remaining six patients.

**Conclusions:**

Rescue RFA/PEI may provide an alternative to trigger further growth of the FLR in patients with cirrhosis-related HCC showing insufficient FLR after RALPPS stage-1.

*Trial registration* Retrospectively registered.

## Introduction

Portal vein embolization (PVE) or ligation (PVL) is a standard procedure to stimulate liver growth in patients planned for extended hepatectomy but with insufficient future liver remnant (FLR). A 27–39% increase of FLR can be achieved within 4–8 weeks, but in 25%–38% of the patients hepatectomy is not possible due to tumor progression or insufficient FLR [1]. In 2012, a novel two-staged hepatectomy strategy where in stage-1 PVL was combined with a liver parenchyma transection between the FLR and the deportalized lobes, followed by hepatectomy in stage-2 emerged as a promising technique for patients with insufficient FLR [2]. The procedure was then named ALPPS (associating liver partition and portal vein ligation for staged hepatectomy) [3]. Compared with solely performing PVE or PVL, the additional liver parenchyma transection in ALPPS produces a dramatic effect: it can generate rapid FLR growth (40–160%) in a short time (6–9 days) [4], allowing a higher completion rate of liver tumor resection (95–100%) [5]. Interestingly, in cases of failed growth after standard PVE, supplementary liver parenchyma transection can still trigger FLR growth, increasing the resectability rate of liver tumors (so-called rescue ALPPS) [6, 7].

However, the transection of the liver parenchyma during ALPPS stage-1 results in a high risk of complications such as biliary leakage. The morbidity and mortality of ALPPS was reported as high as 68% and 12% respectively in the initial report [2], which sparked criticism concerning its actual benefit. To minimize the invasiveness of ALPPS many technical variants have been proposed. One of those techniques is RFA-assisted ALPPS (RALPPS), open or under laparoscopy [8]. Instead of surgical transection it uses radiofrequency ablation (RFA) to create an avascular plane between the deportalized lobes and the FLR. Previous researches have demonstrated that RALPPS can induce a similar FLR increase as that of classic ALPPS [9], while reducing the perioperative morbidity and mortality [9, 10].

Presently, ALPPS or its variants are mainly performed in patients with colorectal liver metastases (CRLM), whereas few studies focus on hepatocellular carcinoma (HCC) (64% versus 14% according to the international ALPPS registry www.alpps.net) [11]. The major concerns about HCC include the poor liver function reserve associated with HCC, due to chronic liver inflammation and compromised growth capacity affected by liver fibrosis/cirrhosis. Therefore, the inclusion criteria for HCC patients to undergo ALPPS is stricter requiring a higher percentage of FLR (≥ 40% versus 25–30% in normal liver) to ensure a safe hepatectomy [12, 13]. Applying that threshold, RALPPS has been shown to be a safe and efficient technique in patients with cirrhosis-related HCC [10].

However, even with a strict selection of HCC patients for ALPPS or its variants, the FLR still faces the risk of poor growth. For these failed cases, the general treatment in clinical practice is palliative therapy such as transarterial chemoembolization (TACE) and/or sorafenib. At our center, we have developed a technique to trigger further growth of the FLR in those patients by applying supplemental percutaneous RFA or percutaneous ethanol injection (PEI). These are two minimally invasive, repeatable procedures commonly used in treatment of liver tumors. We coined this strategy as “rescue RFA/PEI”. In the present study, we report the initial results of this strategy, interpret the possible mechanism and discuss its potential implications for extended hepatectomy.

## Methods

Clinical data was retrospectively collected for all patients who underwent supplemental percutaneous RFA or PEI after RALPPS stage-1 at the Southwest Hospital of Army Medical University, China, between September 2014 and October 2016. RALPPS was performed in patients clinically diagnosed with HCC according to the criteria published by the American Association for the Study of Liver Disease [14], insufficient FLR volume (FLR < 40% in fibrosis/cirrhosis cases), Child–Pugh scoring A, an indocyanine green retention rate at 15 min (ICG-R15) less than 10%. The metastasis in extrahepatic organs was ruled out by positron emission tomography-CT if necessary. The viral load of hepatitis B virus (HBV) was decreased to 10^4^ level (IU/ml) with antiviral treatment before undergoing the RALPPS, if any active hepatitis.

This study was performed in accordance with the Helsinki Declaration. The research protocol was approved by the Southwest Hospital Ethics Committee Hospital Ethics Committee (No.KY2020177) and written informed consent was waived from all patients due to the nature of a retrospective study.

### Liver volume assessment

Baseline FLR volume and its dynamic changes after RALPPS stage-1 and rescue RFA/PEI were obtained from the clinical contrast-enhanced computed tomography (CT) scan using the software Amira 4.1 (VSG Inc., Hillsboro, OR) by a technician (YJ.J) with 10 years of experience of upper abdominal CT imaging evaluation. The standardized FLR was defined as: FLR volume/standardized total liver volume (sTLV). sTLV was calculated according to a formula: 706.2 * body surface area (m^2^) + 2.4 [15]. The FLR increase was calculated as: (FLR vol_post_^–^FLR vol_0_)/FLR vol_0_*100%, in which FLR vol_0_ stands for the baseline FLR volume and FLR vol_post_ refers to the FLR volume after stage-1. Kinetic growth rate (KGR) was expressed in two forms of absolute KGR (ml/day) and relative KGR (%/week) [16].

### Surgical technique for the RALPPS stage-1

Under general anesthesia, an inverted L-shaped subcostal incision was made in the right upper quadrant. An intraoperative ultrasound was performed in all patients to assess actual tumor situation. After cholecystectomy, the right hepatic pedicle was isolated without mobilizing the liver. Following the ligation of the corresponding branch of the portal vein, a demarcation line would become distinct. Then, using an RFA device (Habib 4X, RITA 4401L, AngioDynamics Inc., Manchester, GA, U.S.), the liver tissue was cauterized along the demarcation line with a depth ranging from 4 to 6 cm, avoiding major intrahepatic pedicles and the liver hilum. An avascular area of approximately 2 cm in width between the FLR and deportalized lobes was formed. The right hepatic artery and right hepatic duct were tagged with an absorbable suture to facilitate identification during the second stage operation, followed by abdominal wall closure.

### Rescue RFA or PEI

**Fig. 1:**
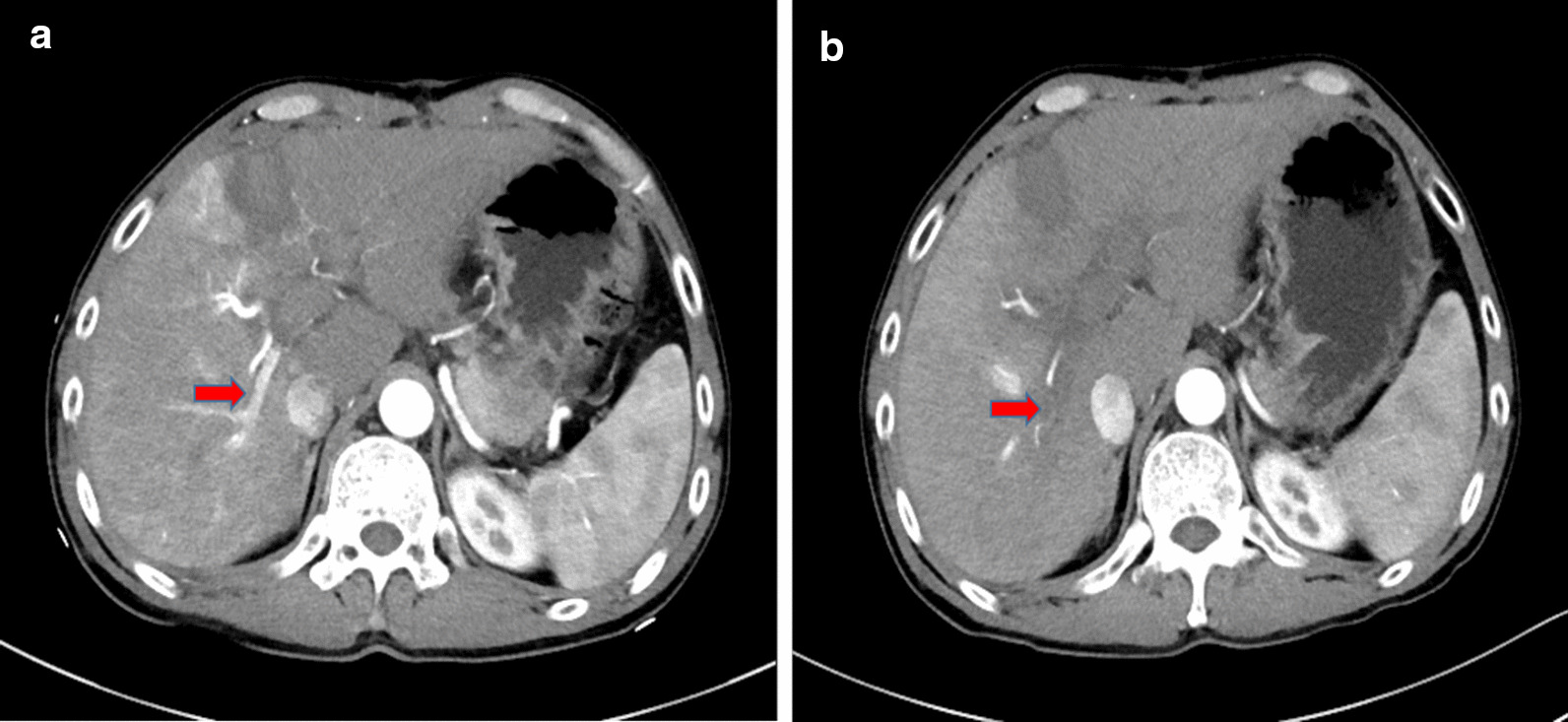
**a** First week after the RALPPS stage-1, ultrasound imaging showed an arterioportal shunt (APS) in the right ligated lobes [red arrow]. Note: RALPPS, radiofrequency-assisted ALPPS (associating liver partition and portal vein ligation for staged hepatectomy). **b** After three sessions of rescue percutaneous ethanol injection (PEI) with 10 mL ethanol each time, the APS disappeared [red arrow]

After RALPPS stage-1, the FLR volume was measured by contrast-enhanced CT at roughly 1-week intervals. If the FLR remained < 40% at the second or third evaluation after RALPPS stage-1, rescue RFA/PEI was considered to induce an artificial damage. All rescue procedures were carried out percutaneously under conscious sedation anesthesia with the guidance of ultrasound. Arterioportal shunts (APS) between the ligated right portal vein and the hepatic artery were diagnosed on the contrast-enhanced CT and ultrasound (CE-US) examinations performed after stage 1 (Fig. 1a). When performing rescue PEI, an ultrasound-guided percutaneous PEI with 10 mL of 99% ethanol injected to the reopened portal vein was performed to abolish the APS (Fig. 1b). The ethanol was injected carefully and slowly at a rate of around 2 mL/min through a PTC-B needle (18 G). For patients without APS, RFA was performed for liver tumors located in the deportalized lobes using LDRF-120S (Lead Electron Corp., Mianyang, Sichuan, China) with a RFA duration of 18–20 min (about 6 min per session, 3 sessions). When carrying out the procedure, an RF electrode was inserted into the center of the tumor with an initial power of 50 W, and it was then stepwise withdrawn 1–2 cm for each session. As rescue RFA was not performed for the purpose of the entire tumor ablation, multiple electrodes or multi-site ablations were not required.

### Surgical technique for the RALPPS stage-2

The prerequisites for RALPPS stage-2 (hepatectomy) were an FLR of at least 40%, Child–Pugh grade A, an ICG-R15 < 10% (or when > 10% an FLR above 50% was required), no systemic infection and no metastasis in the FLR or extrahepatic organs. The RALPPS stage-2 for liver resection was performed using the previous incision. Intraoperative ultrasound was used to rule out metastasis in the FLR. Following identification, and after the absorbable suture was detained, the right hepatic artery, right portal vein and bile duct were transected. Following the anterior approach, the avascular area coagulated by the RFA in the first stage and the right hepatic vein were transected. Then, the right perihepatic ligament was transected to remove the tumor-bearing liver lobes. The abdominal cavity was closed after placement of a drainage tube.

### Perioperative complications and mortality

The Clavien-Dindo classification system was applied to evaluate perioperative complications, in which Grades ≥ IIIb were defined as severe complications [17]. Posthepatectomy liver failure (PHLF) was determined by the “50–50” criteria [18]. All deaths during the hospitalization were taken into mortality, no matter whether they were related to surgical operations or not.

### Statistical analysis

Non-normally distributed data were described as median with ranges. Wilcoxon signed-ranks test was used to evaluate the FLR increase rate before and after rescue RFA/PEI and *p* < 0.05 was considered as a significant level. GraphPad Prism (version 8.0, La Jolla California, USA) was used for the statistical analysis and data visualization.

## Results

A total of seven patients with insufficient regeneration after RALPPS stage-1 received rescue RFA/PEI, accounting for 23% of all RALPPS cases (30 patients) during that period. There were six males, and the median age was 44 (33–52) years. All patients had HBV-related fibrosis/cirrhosis (grade 4–6 according to the Ishak scoring system) [19], with no history of surgery or interventional therapy. All tumors were located in the right lobes except in one patient who had tumors on both sides. The median tumor diameter was 7.8 (3.1–17.0) cm. Postoperative pathological examination confirmed the diagnosis of HCC in all five patients who completed RALPPS stage-2. Detailed preoperative characteristics of the patients and the liver tumor are provided in Tables 1 and 2.

**Table 1:** Patient preoperative characteristics

Patient	Sex	Age (years)	AFP (ng/mL)	Fibrosis grade^a^	Child–Pugh grade and score	ICG-R15 (%)	MELD score
1	M	51	229	6	A (6)	4.4	19
2	M	42	13	6	A (5)	3.9	9
3	M	39	375	6	A (5)	2.4	7
4	M	52	56,330	5	A (5)	3.2	9
5	M	52	47,340	6	A (6)	1.2	4
6	M	44	2100	4	A (5)	2.8	5
7	F	33	4910	5	A (5)	1.9	4
Summary	6:1(M:F)	44 (33–52)	2100 (13–56330)	–	–	2.8 (1.2–4.4)	7 (4–19)

^a^Classified according to the Ishak scoring system; AFP: alpha fetoprotein; ICG-R15, indocyanine green test retention rate at 15 min; MELD, model for end-stage liver disease. The summary data is expressed as median with ranges

**Table 2:** Tumor characteristics

Patient	Location	Number	Size (cm)^a^	PVTT	HVTT	Pathology^b^
1	Right anterior sector	1	7.8	No	No	Moderately differentiated HCC
2	V, VI	Multiple	7.0	No	Right hepatic vein	Poorly differentiated HCC
3	VI	1	6.8	Right branch	No	–
4	Right hemi-liver	2	10.1	Right posterior branch	No	Moderately differentiated HCC
5	Right and left lobes	3	17.0	Right branch	Right hepatic vein, middle hepatic vein	–
6	V, VIII	1	11.6	No	No	Moderately differentiated HCC
7	V, VI, VII	Multiple	3.1	No	No	Moderately differentiated HCC

^a^Refers to the maximum diameter of the tumor; ^b^Two patients did not complete the RALPPS stage-2 and had no pathological diagnosis; RALPPS indicates radiofrequency-assisted ALPPS (associating liver partition and portal vein ligation for staged hepatectomy); PVTT, portal vein tumor thrombus; HVTT, hepatic vein tumor thrombus; HCC, hepatocellular carcinoma

### Volumetric change of FLR

The median baseline FLR volume was 339 ml (225–420), corresponding to 26% (19–33%) of sTLV. The first week after RALPPS stage-1 induced the highest FLR growth rate, with a median KGR of 10% (− 1% to 15%), after that the KGR dropped significantly to 1.5% (0 to 9%) during the second week (p < 0.05). FLR increased to 38% (27–44%) before the rescue RFA/PEI was applied.

**Fig. 2:**
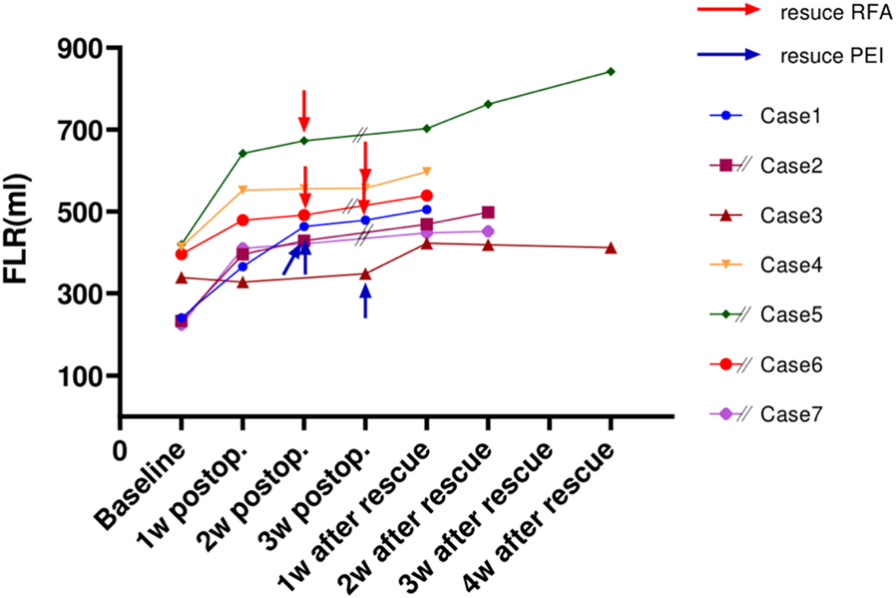
Dynamic volume changes of the future liver remnant (FLR) after RALPPS stage-1 and rescue RFA/PEI procedures. Red or blue arrows stand for the starting of rescue procedures. PEI indicates percutaneous ethanol injection; postop., postoperative; RFA, radiofrequency ablation; RALPPS, RFA-assisted ALPPS (associating liver partition and portal vein ligation for staged hepatectomy). [Note: the time scale has been adjusted. In patient 1,3,4 the rescue was performed 3 weeks post stage-1 and in the others it was done 2 weeks post stage-1, but the liver volume is shown at the same time-point 1 week after rescue RFA/PEI]

Five sessions of rescue RFA and eight sessions of rescue PEI were performed in total, in which one session of RFA was performed on three patients, two sessions of RFA on one patient, and one, three and four session(s) of PEI on the remaining three patients. The patients experienced a second rapid growth of FLR in the first week after rescue procedures (median KGR 4%, ranging from 2 to 5%). There was a significant difference in KGR between before and after rescue procedures (p < 0.05). Five patients finally succeeded with the RALPPS stage-2 with a safe FLR level of 41% (40–47%), corresponding to a volume of 505 ml (452–597). The median FLR increase was 101% (36–113%) or 227 ml (143–264), during a median interval period of 27 days (21–32) (Tables 3, 4, Fig. 2).

**Table 3:** Volumetric changes of the FLR

Patient		Baseline	1st week after stage-1	2nd week after stage-1	3rd week after stage-1	1st week after rescue	2nd week after rescue	4th week after rescue	Before stage-2
1	FLR (ml)	241	366	463	479	505^Ra^	–	–	505
	FLR (%)	19	29	37	38	41	–	–	41
2	FLR (ml)	234	395	429	–	469 ^P,Pb^	498^P^	–	498
	FLR (%)	19	32	35	–	38	41	–	41
3	FLR (ml)	339	327	–	348	422^P,P^	419	412^P,P^	–
	FLR (%)	26	26	–	27	33	33	32	–
4	FLR (ml)	414	552	556	557	597^R^	–	–	597
	FLR (%)	33	44	44	44	47	–	–	47
5	FLR (ml)	420	642	673	–	702^R^	762^R^	842	–
	FLR (%)	26	39	40	–	43	47	52	–
6	FLR (ml)	396	479	491	–	539^R^	–	–	539
	FLR (%)	29	35	36	–	40	–	–	40
7	FLR (ml)	225	410	421	–	448^P^	452	–	452
	FLR (%)	21	38	39	–	42	42	–	42
1	KGR (ml/day)		17.8	16.3	2.0	5.1	–	–	
	KGR (%/week)		10	9	1	3	–	–	
2	KGR (ml/day)		26.9	4.8	–	8.1	5.8	–	
	KGR (%/week)		15	3	–	5	3		
3	KGR (ml/day)		− 1.2	–	2.3	8.3	− 0.5	0	
	KGR (%/week)		− 1	–	1	5	0	0	
4	KGR (ml/day)		17.3	0.4	0.3	5.0	–	–	
	KGR (%/week)		10	0	0	3	–	–	
5	KGR (ml/day)		31.7	4.5	–	4.2	7.5	3.6	
	KGR (%/week)		14	2	–	2	3	2	
6	KGR (ml/day)		13.9	1.5	–	9.6	–	–	
	KGR (%/week)		7	1	–	5	–	–	
7	KGR (ml/day)		23.2	1.5	–	6.3	0.5	–	
	KGR (%/week)		15	1	–	4	0	–	

^a^R indicates rescue radiofrequency(RFA); ^b^P, rescue percutaneous ethanol injection(PEI). The number of subscript R or P stands for the number of session(s) applied; FLR, future liver remnant; KGR, kinetic growth rate

**Table 4:** Summary of the FLR changes

Variable	Median (range)
Baseline	
FLR, ml	339 (225–420)
FLR, %	26 (19–33)
First week after RALPPS stage-1	
KGR, ml/day	17.8 (− 1.2–31.7)
KGR, %/week	10 (− 1–15)
Second week after RALPPS stage-1	
KGR, ml/day	3 (0.4–16.3)
KGR, %/week	1.5 (0–9)
Before rescue procedures	
KGR, ml/day	2.0 (0.3–4.8)
KGR, %/week	1 (0–3)
After rescue procedures	
KGR, ml/day	6.3 (4.2–9.6)
KGR, %/week	4 (2–5)
Interval period, days^a^	27 (21–32)
Before RALPPS stage-2^a^	
FLR, ml	505 (452–597)
FLR, %	41% (40–47%)
Increase rate^a^	
FLR, ml	227 (143–264)
FLR, %	101% (36–113%)
KGR, ml/day	9.4 (5.7–9.9)
KGR, %/day	3.9 (1.4–4.4)
Completion rate	5/7 (71%)

FLR, future liver remnant; KGR, kinetic growth rate; RALPPS, radiofrequency-assisted ALPPS (associating liver partition and portal vein ligation for staged hepatectomy); ^a^Five patients completed RALPPS stage-2

### Perioperative complications and mortality

There were no rescue RFA/PEI related complications. One patient experienced in-hospital mortality due to renal failure and severe pulmonary infection after stage-2 of hepatectomy, otherwise there were no other severe complications (Grades ≥ IIIb).

Insufficient FLR growth was observed in two patients. For one patient, the FLR even diminished during the first week after RALPPS stage-1 and was then stationary in size (27%). The rescue PEI promoted a slight growth of FLR but then the FLR fluctuated at the level of 33% even after four sessions of PEI were applied. For the other patient, although a sufficient FLR was achieved immediately after RALPPS stage-1, the liver function worsened (ICG-R15 > 10%) which contraindicated the invasive liver resection. With additional rescue RFA, the FLR eventually increased to 52%. However, during the period of volume enhancing procedures a metastasis was developed in the FLR, detected at the final volumetric liver CT scan. As RALPPS stage-2 was no longer an option both patients underwent further interventional therapy.

### Intraoperative data

One patient received intraoperative RFA for a tumor in the FLR during RALPPS stage-1. Laparoscopic RALPPS stage-1 was performed in one case. For all seven patients, the median operation time was 195 min (170–278), with a median intraoperative RFA time of 10 min (8–30). The median blood loss was 100 mL (50–400) without red blood cell transfusion in any cases.

Of the five patients who finally proceeded to the stage-2 two underwent a right trisectionectomy and three right hepatectomy. At stage-2 the blood loss was 350 ml (200–600), and the operation time was 274 min (210–411). No RBC transfusion was applied during this stage. All surgical resection margins were histopathologically negative (R0 resection). Detailed intraoperative data are given in Table 5.

**Table 5:** Intraoperative and interstage period data

Patient	RALPPS stage-1			Interstage period			RALPPS stage-2	
	Blood loss (ml)	Operation time (min)	Intraoperative RFA time (min)	Interstagen period (days)	Rescue RFA session (s)	Rescue PEI session (s)	Blood loss (ml)	Operation time (min)
1	300	180	15	28	1	–	360	210
2	100	270	10	27	–	3	350	286
3	100	195	14	–	–	4		
4	50	278	8	32	1	–	200	274
5	400	277	30	–	2	–		
6	200	170	8	21	1	–	600	273
7	50	188	10	23	–	1	300	411
Summary	100 (50–400)	195 (170–278)	10 (8–30)	27 (21–32)	1 (1–2)	3 (1–4)	350 (200–600)	274 (210–411)

FLR, future liver remnant; PEI, percutaneous ethanol injection; RFA, radiofrequency ablation; RALPPS, RFA-assisted ALPPS (associating liver partition and portal vein ligation for staged hepatectomy). The summary data is expressed as median with ranges

## Discussion

To the best of our knowledge, this is the first report on rescue RFA/PEI to promote further hypertrophy of the FLR after failed RALPPS stage-1. According to this retrospective single center experience in seven patients further FLR growth can be induced by applying percutaneous RFA or PEI.

Until now, the exact mechanisms behind the fast and powerful FLR hypertrophy in ALPPS remain unclear. Most researchers agree that the rapid hypertrophy of the FLR is probably a combined consequence from inflammatory mediators and growth factors caused by surgical trauma during parenchyma transection [20]. These are further enriched in the FLR by the ligation of the right portal vein, directing mediators, growth factors, and bowel nutrients into the FLR. Also, the parenchymal transection may prevent eventual portal venous shunting via potential collaterals between the FLR and deportalized lobes [3; 20]. In an experimental study it has been shown that plasma from mice undergoing ALPPS mice injected into PVL mice increases FLR to the same extent as if undergoing ALPPS [21]. Interestingly, in the same study, the researchers damaged spleen, kidney and lung of mice in another group, using bipolar ablation to create a local inflammation and yielded a similar hypertrophy of the FLR after PVL as in the ALPPS group [21]. Moreover, it has been shown that partial (50%–80%) transection of the liver parenchyma in patients undergoing ALPPS stage-1 (partial ALPPS) can also achieve rapid hypertrophy of the FLR [22]. Our study illustrates that the induced inflammation from rescue RFA/PEI can further stimulate FLR increase after the first growth induced by the stage-1 trauma.

After the ligation of the right portal vein branch at stage-1, the hemodynamic change in liver is dramatic. With the increased blood volume and pressure in the unligated portal vein branch collaterals between the liver lobes may reopen. The development of APS is one example of such reopening collaterals. Those often occur in the cirrhotic liver, especially after PVL [23]. APS to the residual right portal branch after classic ALPPS stage-1 has been described [24] and by elimination of intrahepatic APS further FLR growth can be induced. In the present study, an APS was developed in three patients during the interstage period [25]. As its existence may undermine the hypertrophy of the FLR, additional PEI was applied to obliterate the APS. In the setting of rescue RFA, the tumor in the ligated lobe was ablated to trigger an artificially inflammatory response. The hypothesis was that resultant inflammatory cytokines may contribute to the FLR growth. This hypothesis was based on results from animal studies, where the observed liver regeneration after RFA was considered to be caused by the global inflammatory response it induced [26, 27].

Many researchers have questioned that the augmented FLR in the short time after stage-1 (median 9 days in classic ALPPS) is not a functional regeneration [28]. Hepatocytes in the FLR tissue have been shown to be immature at histological analysis 11 days after stage-1 [29]. Technically, waiting for at least 2–3 weeks after stage-1 may ensure a safe extended hepatectomy, particularly vital for cirrhosis-related HCC where the hepatocytes are less functional already at baseline. On the other hand, as liver growth after operation experiences a fast-to-slow process, we assumed that if the FLR could not reach the target level in 2–3 weeks after RALPPS stage-1, its possibility to reach a safe level in the following time is low. That is the reason why we applied rescue RFA/PEI during the second or third week after stage-1. Still, the ideal timing to perform rescue procedures needs to be specified further.

The surgical trauma at cirrhosis-related HCC must be minimized due to limited liver functional reserve. As no traumatic surfaces are formed at RALPPS, the risk of biliary leakage or intraperitoneal infection is reduced. It has been demonstrated that the morbidity is mainly caused by biliary leakage [30–32]. Also, the intraoperative blood loss and abdominal adhesions are reduced in RALPPS stage-1. A report from the ALPPS registry comprising 202 subjects showed that blood transfusion in ALPPS stage-1 is an independent risk factor for severe complications [32]. Collectively, these improvements provided by RALPPS create a good condition for the second stage of hepatectomy. In this study, none of the seven patients showed any major complication or sign of PHLF.

In the present study, there was one patient who failed to undergo hepatectomy even though four sessions of rescue PEI were performed. After RALPPS stage-1, the FLR volume unexpectedly diminished slightly and then maintained at that level. This finding implies that plenty of factors influence FLR growth in patients with liver cirrhosis and that sufficient FLR volume augmentation cannot be guaranteed. Future research is warranted to explore the mechanisms affecting liver regeneration in cirrhotic patients.

One patient had a huge, bilateral HCC and received intraoperative RFA for a lesion in the FLR. After rescue RFA, the FLR grew to a safe level for liver resection, but the preoperative CT scan detected metastases in the FLR. Therefore, the second stage operation had to be abandoned. A concern has been raised since the ALPPS introduction that the dramatic increase of blood inflow to the FLR may promote the tumor progression [33]. After that other conclusions have been drawn by different researchers [34, 35]. This controversy may continue with the preexisting debate concerning the oncologic outcomes after conventional PVE/PVL [36]. In this case, we think that for patients with huge HCC and the lesions located in bilateral lobes, the operation decision of ALPPS or its variants should be made with caution.

There was one death in this study. That patient had a history of chronic renal dysfunction during preoperative assessment. After systemic evaluation and discussions within the hospital multidisciplinary team, the patient was regarded as a potential candidate for RALPPS. The patient underwent RALPPS stage-1 uneventfully and his renal function transiently improved during the interstage period. However, after stage-2 his condition deteriorated and progressed to acute renal failure. A severe lung infection also occurred, which in combination with the renal failure caused the patient’s death. Since this patient the inclusion criteria for subsequent patients has been restricted to patients without chronic kidney disease.

There are limitations to be acknowledged in this initial report of rescue RFA/PEI procedure. First, this study is limited by its small sample size. The actual number for rescue RFA and rescue PEI was only four and three patients, respectively. Future research with more participants and controlled groups is warranted to confirm the efficacy of this strategy. Second, as the patients who underwent each rescue procedure was limited, it was not possible to compare their efficiency. Third, the timing to perform rescue procedures needs to be further specified, balancing the sufficient FLR hypertrophy and the risk of tumor progression. Four, the rate of “failed” RALPPS stage-1 was a little high (23%). However, it should be interpreted in the context that a majority of RALPPS patients were accompanied with cirrhosis (c.a. 70%). Finally, this initial study is focused on the safety and efficacy of rescue RFA/PEI, but further studies are needed to show the medium- and long-term outcomes.

## Conclusions

The present study indicates that rescue RFA/PEI can potentially be applied as a strategy to induce FLR further hypertrophy after failed RALPPS stage-1 in patients with liver cirrhosis.

## Data Availability

The datasets used and/or analysed during the current study available from the corresponding author upon reasonable request.

## Abbreviations

FLR: Future liver remnant
HCC: Hepatocellular carcinoma
RALPPS: Radiofrequency-assisted ALPPS
ALPPS: Associating liver partition and portal vein ligation for staged hepatectomy
RFA: Radiofrequency ablation
PEI: Percutaneous ethanol injection
KGR: Kinetic growth rate
PVE/PVL: Portal vein embolization or ligation
CRLM: Colorectal liver metastases
TACE: Transarterial chemoembolization
ICG-R15: Indocyanine green retention rate at 15 min
HBV: Hepatitis B virus
CT: Computed tomography
sTLV: Standardized total liver volume
APS: Arterioportal shunt
PHLF: Posthepatectomy liver failure
